# The effect of cancer on traditional, complementary and alternative medicine utilization in Korea: a fixed effect analysis using Korea Health Panel data

**DOI:** 10.1186/s12906-022-03614-0

**Published:** 2022-05-18

**Authors:** Dongsu Kim, Soo-Hyun Sung, Seungwon Shin, Minjung Park

**Affiliations:** 1 College of Oriental Medicine, Dongshin University, Naju, Jeollanam-do, 58245 South Korea; 2 Department of Policy Development, National Institute of Korean Medicine Development, Junggu, Seoul, 04561 South Korea; 3 National Agency for Korean Medicine Innovative Technologies Development, National Institute of Korean Medicine Development, Junggu, Seoul, 04561 South Korea

**Keywords:** Traditional & Complementary and Alternative Medicine, Traditional Korean Medicine, Korea Health Panel, Cancer occurrence

## Abstract

**Background:**

Cancer-related incidence and mortality rates are rapidly increasing worldwide. However, no studies have examined the effect of cancer as a single factor on the use of traditional, complementary, and alternative medicine (T&CAM). We aimed to determine the effect of cancer occurrence on T&CAM utilization using Korea Health Panel (KHP) data.

**Methods:**

We analyzed longitudinal data (49,380 observations) derived from 12,975 Korean adult participants with complete KHP data from 2011 to 2014 and 2016, and divided them into two groups based on cancer diagnosis. A panel multinomial logit model was used to assess whether the participants used T&CAM or conventional medicine or both in outpatient settings. Additionally, a negative binomial regression model was used to examine the effect of cancer on the number of outpatient visits for T&CAM.

**Results:**

In total, 25.54% of the study participants in the cancer group used T&CAM, which was higher than that in the non-cancer group (18.37%, *p* < 0.0001). A panel multinomial logistic regression analysis using KHP data showed that cancer occurrence was significantly more likely to be associated with ‘Using both Korean medicine and conventional medicine’ (Coef. = 0.80, *p* = 0.017) and ‘Not using Korean medicine but using conventional medicine’ (Coef. = 0.85, *p* = 0.008) than ‘Not using Korean medicine and conventional medicine.’ A panel negative binomial regression showed a significant effect of cancer on increasing the number of T&CAM outpatient visits (Coef. = 0.11, *p* = 0.040).

**Conclusions:**

Our findings showed that cancer occurrence within an individual led to the simultaneous use of conventional medicine and T&CAM. In addition, the occurrence of cancer significantly increased the number of T&CAM outpatient visits among participants already using T&CAM. It was also found that T&CAM has been utilized more often by the most vulnerable people, such as medical beneficiaries and those with a low level of education.

## Background

Cancer is a chronic health condition, and the incidence rate of cancer and cancer-related mortality rates are rapidly increasing globally [1]. It was estimated that 18.1 million new cases of cancer and 9.6 million cancer-related deaths occurred worldwide in 2018 [2]. The principal strategies of cancer treatment include surgical resection, radiotherapy, and chemotherapy [1]; however, increasing numbers of patients with cancer have been choosing traditional, complementary, and alternative medicine (T&CAM). T&CAM is not considered a type of conventional medicine and is based on the methods that different cultures use for prevention and treatment of diseases [3–5]. In a systematic review of 148 studies published in 18 countries, the prevalence of T&CAM use was approximately 40% [6].

T&CAM practitioners in East Asian countries [1, 7], using traditional Chinese medicine, Kampo medicine, or traditional Korean medicine, have attempted to compensate for drawbacks in conventional medicine through increasing patients’ self-defense mechanisms [8]. This includes controlling cancer-related symptoms [9, 10], improving quality of life (QOL), or preventing tumor metastasis [11] using herbal medicine, acupuncture, moxibustion, or qi-gong [9–12].

Several studies have investigated the current status of or related factors in the use of T&CAM in cancer treatment. Berretta et al. conducted a multi-center survey in Italy and reported that 50% of 468 patients with cancer had used T&CAM [13]. A cohort study involving prostate cancer survivors analyzed the socio-demographic, clinical, health-related quality-of-life (HRQOL), and/or psychological factors related to their use of T&CAM therapies [14]. Jones et al. [15] undertook a literature review to determine the clinical, psychological, socioeconomic, or cultural factors that contributed to use of T&CAM by a patient with cancer in Australia. Two cross-sectional studies undertaken in the United States and in South Korea reported that the patients’ positive attitudes toward T&CAM influenced their use of this methodology [16, 17].

However, no studies have yet examined the causal relationship between cancer occurrence and T&CAM utilization. Although randomized controlled trials are viewed as the gold standard to confirm causal relationship, this design may not be feasible to investigate the pure effect of an intervention. Quasi-experimental design can be a solution, using several econometric techniques like Regression Discontinuity (RD), Difference-in-difference (DiD), and Fixed-Effects Model (FE) [18]. This study aimed to determine the effect of cancer occurrence on T&CAM utilization based on a fixed effect model approach using Korea Health Panel (KHP) data.

## Methods

### Data source

KHP data are collected from both the Korea Institute for Health and Social Affairs (KIHASA) and the Korean National Health Insurance Service. KHP data is panel type data relying on an initial random sample of households or individuals which is randomly selected and subsequently followed over time [19]. Reflecting both the cross-sectional (between individuals) and timeseries elements, panel data are also referred to as ‘cross-sectional time-series’ data. A KHP data sample was selected from 2005 Korean Population and Housing Census data using region stratification variables and have been tracked annually since 2008. KHP data reports the medical utilization of inpatient, outpatient, and emergency departments, and it is provided through data files on the use of medical care, household information, household members’ information, and additional investigation.

For this study, we used data from KHP beta version 1.5.1 [16]. We combined KHP data by year to conduct panel analysis and household, household members’, and outpatient medical service usage along with additional investigation files were reorganized and merged to create the final dataset. Year-by-year data were available for a period of five years (2011, 2012, 2013, 2014, and 2016). Data for other years were excluded because the EuroQol-5 Dimension (EQ-5D) variable was unavailable.

### Sample selection

The total number of observations in the five-year KHP data was 70,852. First, we narrowed our target sample to those aged 20 years and older, as this age group was considered capable of making their own decisions in terms of whether they should use conventional medicine or T&CAM. Therefore, we excluded participants under the age of 20 (15,326 observations) from the five-year KHP data. Second, participants who answered “I don’t know” or those who did not answer any question (6,146 observations) were excluded. Finally, 49,380 observations (12,975 participants) were included in this study. Of these, 2,999 observations (1,044 participants) reported that they had been diagnosed with cancer and 46,381 observations (11,931 participants) reported that they had no diagnosis of cancer.

### Variables

#### Dependent variables

To investigate the effect of cancer on T&CAM use, we analyzed outpatient utilization pattern and the number of T&CAM outpatient visits. Outpatient utilization pattern is analyzed over a past 12-month period is categorized into four types: ‘Using both Korean medicine and conventional medicine’, ‘Not using Korean medicine but using conventional medicine’, ‘Not using conventional medicine but using Korean medicine’, ‘Neither using Korean medicine nor conventional medicine’. The number of T&CAM outpatient visits was then calculated using the sum of the number of visits over 12 months.

#### Explanatory variable

The explanatory variable in this study was whether a participant had been diagnosed with cancer or not. When a participant was asked whether he/she had a chronic disease and the participant indicated that he/she had a neoplasm (Korean Standard Classification of Diseases code number: C00-D48), the participant was considered to have cancer for the year in which he/she provided the response.

#### Covariates

Andersen proposed an initial behavioral model to assess how predisposing characteristics, enabling resources, and needs influence the use of health services [20]. Predisposing variables included age, education, and area of residence. Sex, as a time-invariant demographic variable, was not included as it was omitted during the fixed model analysis. The residential areas were divided into rural and urban areas. Urban areas included eight metropolitan cities, and rural areas included nine provinces. Enabling variables included household income (adjusted according to the number of household members) and the type of medical health coverage. Needs factors included specific chronic disease (musculoskeletal, neurological, circulatory, and respiratory diseases) and QOL (EQ-5D). EQ-5D is one of the validated scales to measure QOL, which contains 5 health-related dimensions (mobility, self-care, usual activities, pain/discomfort, and anxiety/depression). Each item can be assessed by 3 levels of responses (no problem, some problems, and extreme problems) [21]. The composite index of EQ-5D were calculated with South Korean population-based preference weights [21]. As EQ-5D is considered one of the need factors, we used previous year’s EQ-5D value.

### Analysis

To analyze participants’ characteristics, we first pooled all the years of the data and conducted a descriptive analysis for each variable. For each variable, the dependent variables such as the outpatient utilization pattern and the number of T&CAM outpatient visits were analyzed. Moreover, the distribution of cancer occurrence, the main explanatory variable, was also presented. All the continuous variables were analyzed using t-test or analysis of variance (ANOVA) test, while nominal variables were analyzed using chi-square test.

We conducted multivariate regression analyses to determine whether cancer occurrence had an effect on the use of T&CAM and how many times T&CAM was used. Panel analysis was performed according to the characteristics of the repeated measured data, which was used to overcome the endogenous problem caused by the correlation between unobserved individual characteristics and health care utilization [22]. It is known that panel analysis mainly uses a fixed-effect model and a random-effect model. These models are differentiated according to how unobserved individual characteristics are addressed within the error term [23, 24]. The fixed-effect model deletes the differences between individuals while removing unobserved characteristics [24]. Therefore, it only targets differences within the same individual unit. However, the random-effect model addresses unobserved differences between individuals as a probability which has a distribution, rather than deleting them [24]. For this study, we used a fixed-effect model to examine the effect of cancer occurrence on the use of T&CAM, whereas other demographics, which were either observed or not, were controlled by using within-individual variation.

We employed two models for the analysis based on the characteristics of the dependent variables. First, we used a panel multinomial logit model to determine whether the participants used T&CAM, conventional medicine, or both in outpatient settings. A logit model is used to determine discrete distributions through answering “Yes” or “No”. However, if there were more than three selectable items for the dependent variable, a multinomial logistic regression analysis was used. We organized the dependent variables into four types based on the outpatient utilization pattern, which are ‘Using both Korean medicine and conventional medicine’, ‘Not using Korean medicine but using conventional medicine’, ‘Not using conventional medicine but using Korean medicine’, ‘Neither using Korean medicine nor conventional medicine’. Second, a negative binomial regression model was used to examine the effect of T&CAM on the number of outpatient visits. A negative binomial model is used to resolve issues concerning the overdispersion of Poisson regression analysis, which also considers the number of occurrences. All analyses were conducted using the Stata software (Stata SE, version 17.0 Stata Corp, College Station, TX, USA). The significance level for the verification of the hypotheses was set at 0.05.

## Results

### Demographics

The baseline characteristics of the study participants according to the occurrence of cancer are presented in Tables 1 and 2. Cancer observations comprised 6.07% of the total observations (Table 1). Of these, 25.54% used T&CAM, which was significantly higher than the number of participants without cancer who used T&CAM (18.37%) (*P* < 0.0001). The average number of times a participant with cancer used T&CAM was 2.86 times, which was significantly higher than that of participants without cancer who used T&CAM (1.94) (*p* < 0.0001). Of the participants who used conventional medicine, 21.56% also used T&CAM; however, there was a significantly lower percentage of participants (3.56%) who used only T&CAM (and no conventional medicine) (Table 2).

**Table 1:** Number of participants and cancer occurrence by year

Year	2011 (*n* = 11,109)		2012 (*n* = 10,443)		2013 (*n* = 9,796)		2014 (*n* = 9,316)		2016 (*n* = 8,716)		Total number of participants (*n* = 49,380)	
Participants	n	%	n	%	n	%	n	%	n	%	n	%
Cancer occurrence												
Yes	613	5.52	605	5.79	623	6.36	600	6.44	558	6.40	2,999	6.07
No	10,496	94.48	9,838	94.21	9,173	93.64	8,716	93.56	8,158	93.60	46,381	93.93

**Table 2:** Descriptive characteristics of participants, Cancer occurrence, outpatient utilization pattern

Variables	Total (*N* = 49,380)	Cancer Occurrence^a^ (% or SD)		
		No	Yes	*p*-value
T&CAM use^a^				
Nonuser	46,381	37,861 (81.63)	8,520 (18.37)	< 0.0001***
User	2,999	2,233 (74.46)	766 (25.54)	
# of T&CAM use ^b^		1.94 (8.29)	2.86 (10.70)	< 0.0001***
Conventional medicine use				
Nonuser	7,563	7,494 (99.09)	69 (0.91)	< 0.0001***
User	41,817	38,887 (92.99)	2,930 (7.01)	
Control variable				
Age (Year)		52.54 (16.51)	59.41 (13.34)	< 0.0001***
Education				
Elementary or lower	11,348	10,497 (92.50)	851 (7.50)	< 0.0001***
Middle school or high school	21,634	20,076 (92.80)	1,558 (7.20)	
University or higher	16,398	15,808 (96.40)	590 (3.60)	
Region				
Urban	21,397	20,087 (93.88)	1,310 (6.12)	0.690
Rural	27,983	26,294 (93.96)	1,689 (6.04)	
Household income ^c^ (10,000 won)		676.616 (520.15)	722.88 (585.35)	< 0.0001***
Health care coverage				
National health insurance	49,928	44,166 (94.11)	2,762 (5.89)	< 0.0001***
Medical benefits	1,993	1,823 (91.47)	170 (8.53)	
special cases	459	392 (85.40)	67 (14.60)	
Chronic musculoskeletal diseases				
No	32,549	30,923 (95.00)	1,626 (5.00)	< 0.0001***
Yes	16,831	15,458 (91.84)	1,373 (8.16)	
Chronic neural diseases				
No	47,296	44,500 (94.09)	2,796 (5.91)	< 0.0001***
Yes	2,084	1,881 (90.26)	203 (9.74)	
Chronic circulatory diseases				
No	35,030	33,119 (94.54)	1,911 (5.46)	< 0.0001***
Yes	14,350	13,262 (92.42)	1,088 (7.58)	
Chronic respiratory diseases				
No	44,873	42,236 (94.12)	2,637 (5.88)	< 0.0001***
Yes	4,507	4,145 (91.97)	362 (8.03)	
Quality of life (EQ-5D)		0.91 (0.09)	0.89 (0.11)	< 0.0001***
Year				
2011	11,109	10,496 (94.48)	613 (5.52)	0.013*
2012	10,443	9,838 (94.21)	605 (5.79)	
2013	9,796	9,173 (93.64)	623 (6.36)	
2014	9,316	8,716 (93.56)	600 (6.44)	
2016	8,716	8,158 (93.60	558 (6.40)	

^a^The results for continuous variables are based on t-tests, and nominal variables based on chi-square tests

^b^t-test or ANOVA used only on nominal variables

^c^The household income corrected by dividing it with the root of the number of household members

^*^: *p* < 0.05, **:*p* < 0.01, ***:*p* < 0.001

### The effect of cancer occurrence on the use of T&CAM

Table 3 shows the effect of cancer occurrence on the use of T&CAM in terms of panel multinomial logistic regression analysis using the KHP data. Cancer occurrence was significantly more likely to be associated with ‘Using both Korean medicine and conventional medicine’ (Coef. = 0.80, *p* = 0.017) and ‘Not using Korean medicine but using conventional medicine’ (Coef. = 0.85, *p* = 0.008) than those under the category of ‘Neither using Korean medicine nor conventional medicine’. However, the probability that cancer occurrence was associated more with ‘Using Korean medicine and not using conventional medicine’ than with ‘Neither using Korean medicine nor conventional medicine’ was not significant (Coef. = -1.05, *p* = 0.381). These findings indicate that the occurrence of cancer causes individual conventional outpatient use but also induces concurrent conventional outpatient use and T&CAM outpatient use.

**Table 3:** The impact of having cancer upon the use of T&CAM based on panel multinomial logistic regression^a^ (*n* = 49,380)

Variables	Using both Korean medicine and conventional medicine		Not using conventional medicine but using Korean medicine		Not Using Korean medicine but using conventional medicine	
	Coef^b^	*p*-value	Coef^b^	*p*-value	Coef^b^	*p*-value
Explanatory variable						
Having cancer						
No (Ref)						
Yes	0.80	0.017*	-1.05	0.381	0.854	0.008**
Control variable						
Age (year)	0.18	0.163	0.63	0.119	-0.12	0.267
Spouse						
No (Ref)						
Yes	0.07	0.807	0.11	0.917	0.35	0.138
Education						
Elementary or lower (Ref)						
Middle school or higher	14.35	0.986	29.14	0.991	15.02	0.985
University or higher	16.73	0.984	44.24	0.991	17.84	0.983
Region						
Urban (Ref)						
Rural	-0.00	0.989	0.89	0.394	0.01	0.979
Household income ^c^ (10,000 won)	-0.00	0.477	0.00	0.907	-0.00	0.315
Health care coverage						
National health insurance (Ref)						
Medical benefits	1.05	0.032*	2.88	0.011*	0.86	0.051
special cases	-0.55	0.329	0.53	0.743	-0.20	0.702
Chronic musculo-skeletal diseases						
No (Ref)						
Yes	0.49	0.004**	1.04	0.029*	0.41	0.010*
Chronic neural diseases						
No (Ref)						
Yes	0.63	0.189	-13.19	0.991	0.65	0.160
Chronic circulatory diseases						
No (Ref)						
Yes	1.21	< 0.0001***	0.71	0.290	1.35	< 0.0001***
Chronic respiratory diseases						
No (Ref)						
Yes	0.49	0.098	1.88	0.156	0.31	0.258
Quality of life (EQ-5D)	1.95	< 0.0001***	1.54	0.414	1.14	0.018*
Year						
2011(Ref)						
2012	0.36	< 0.0001***	0.18	0.405	0.20	< 0.0001***
2013	0.51	< 0.0001***	-0.15	0.530	0.27	< 0.0001***
2014	0.49	< 0.0001***	0.33	0.148	0.37	< 0.0001***
2016	0.48	< 0.0001***	0.15	0.573	0.40	< 0.0001***
Log likelihood	-11,205.353					

^a^Neither using Korean medicine nor using conventional medicine’ of dependent variable is base outcome

^b^Coefficient

^c^The household income corrected by dividing it with the root of the number of household members

^*^: *p* < 0.05, **:*p* < 0.01, ***:*p* < 0.001

Besides cancer occurrence, QOL (EQ-5D) has a significant effect on ‘Using both Korean medicine and conventional medicine’ (Coef. = 1.95, *p* < 0.0001) and ‘Not using Korean medicine but using conventional medicine’ (Coef. = 1.14, *p* < 0.0001) than ‘Neither using Korean medicine nor conventional medicine’. Certain chronic diseases have shown similar results.

### The effect of cancer on the number of T&CAM outpatient visits

Table 4 shows the effect of cancer on the number of outpatient visits for T&CAM using a panel negative binomial regression. As a result, cancer had a significant effect on increasing the number of T&CAM outpatient visits (Coef. = 0.11, *p* = 0.040). In terms of the other variables, participants with medical benefits, chronic musculoskeletal or nervous system diseases, and those with high QOL showed significantly more frequent use of T&CAM than those with national health insurance, without specific chronic diseases, and with low QOL. In contrast, participants with spouses and with middle school or higher education used T&CAM less frequently than those without spouses and with elementary school education.

**Table 4:** Impact of cancer on the number of T&CAM sessions based on Panel negative binomial regression (*n* = 49,380)

Variables	Coef.^†^	p-value
Explanatory variable		
Having cancer		
No (Ref)		
Yes	0.11	0.040*
Control variable		
Age (Year)	0.01	0.831
Spouse		
No (Ref)		
Yes	-0.11	0.009**
Education		
Elementary or lower (Ref)		
Middle school or high school	-0.11	0.020*
University or higher	-0.24	<0.0001***
Region		
Urban (Ref)		
Rural	0.01	0.779
Household income ∮ (10,000 won)	0.00	0.132
Health care coverage		
National health insurance (Ref)		
Medical benefits	0.21	0.008**
special cases	-0.15	0.217
Chronic musculo-skeletal diseases		
No (Ref)		
Yes	0.32	<0.0001***
Chronic neural diseases		
No (Ref)		
Yes	0.12	0.049*
Chronic circulatory diseases		
No (Ref)		
Yes	-0.04	0.314
Chronic respiratory diseases		
No (Ref)		
Yes	0.01	0.922
Quality of life (EQ-5D)	0.38	0.007**
Year		
2011 (Ref)		
2012	0.14	<0.0001***
2013	0.20	<0.0001***
2014	0.11	0.001**
2016	0.11	0.002**
constant	-2.04	<0.0001***
Log likelihood	-24752.256	

^†^Coefficient

^*^: *p* < 0.05, **: *p* < 0.01, ***: *p* < 0.0001

## Discussion

In recent studies, the prevalence of T&CAM use for cancer treatment has been reported to range from 37 to 66%, and T&CAM is widely used as an adjuvant therapy for cancer [1, 6, 25–28]. Those studies were mainly conducted as surveys [13, 17, 26, 29, 30], literature reviews [15, 27], and cross-sectional studies, and were concerned with T&CAM prevalence, intervention, and the type of cancer when applying T&CAM methodologies. However, as the results were derived from cross-sectional surveys of patients with cancer, none of these studies provided information concerning the effect of cancer occurrence on the use of T&CAM over time and how many times T&CAM was used.

When focusing on cancer occurrence and T&CAM use, multinomial panel logistic analysis results showed that cancer occurrence within an individual did not lead to the use of T&CAM alone, but it led to the simultaneous use of conventional medicine and T&CAM. The panel negative binomial regression analysis showed that cancer occurrence had a significant positive effect on the number of T&CAM outpatient visits. The descriptive statistics of this study indicated that the average number of T&CAM utilization among the participants with cancer was 2.86, which was significantly higher than the average number of T&CAM utilization in the non-cancer participant group (1.94). This finding is similar to results of previous reports [13] stating that cancer patients commonly use T&CAM and that most cancer patients use T&CAM with conventional medicine simultaneously.

The analysis of the panel negative binomial regression showed that participants from the Honam region used T&CAM more intensively than those from the Seoul/Gyeonggi region. There are many Korean medicine universities and hospitals in the Honam region, as of 2019 [31]. As such, a cultural atmosphere favorable to Korean medicine and relatively easy access to medical institutions could be the reason for the high frequency of T&CAM utilization.

The two main findings of this study were as follows: (i) cancer occurrence within an individual did not lead to the use of T&CAM alone, but it led to the simultaneous use of conventional medicine and T&CAM; (ii) among participants who were already using T&CAM, the occurrence of cancer significantly increased the number of T&CAM outpatient visits.

Apart from cancer-related use, it was also found that T&CAM were more often used by the most vulnerable people, such as medical beneficiaries and those with a low level of education, than participants with national health insurance and middle school education level or higher (Tables 3 and 4). Medical beneficiaries are determined when recipients’ recognized income is 40% or less of the standard median income in Korea. They are known to use outpatient treatment more than national health insurance subscribers because of their low health-related quality of life [32]. Our research found that not only conventional medicine but also T&CAM is an easily accessible medical service modality for vulnerable people.

This study had some limitations. First, T&CAM is used in the treatment of cancer in South Korea, Japan, and China. Specific treatments include acupuncture, moxibustion, cupping, concoction, and qi-gong [33]. In the United States or in European countries, alternative and supplementary treatments such as herbal medicine, vitamins, massage, mind–body therapy (for example, meditation, relaxation techniques) have been used for patients with cancer [13, 34]. Our study findings are based on representative sample data from South Korea, so they cannot be readily applied to countries with different environments, such as in Europe or the United States. Therefore, in our analysis of whether people with cancer used T&CAM or not and which pattern they used, different health care systems, and the different positions of T&CAM must be considered, and further studies are necessary to verify our findings.

Second, due to limitations in the KHP dataset, it was not possible to obtain information concerning cancer stage or specific types of cancer, which made it challenging to conduct a more in-depth analysis. To develop evidence-based policies regarding cancer, it is necessary to obtain reliable evidence-based data [35]. Future studies need to ensure that detailed questions can be posed in relation to comprehensive datasets to extend the possibility of determining more informative results concerning cancer.

Third, some variables that could have affected medical use were omitted. According to Anderson's model, the need factor has the greatest influence on medical use, which is a disease-related factor. Though we attempted to utilize the number of chronic diseases variables, however, this was not used due to increased multicollinearity with other chronic disease variables. The ‘Chlarson comorbidity index’ was also considered, but since the KCD code was not used in the 2011 data, it could not be applied.

This study confirmed that the use of T&CAM and conventional medicine significantly increased in the event of cancer occurrence. With recent advances in medical technology, the five-year survival rate is increasing [36]. This suggests the need to establish a treatment guide for the combination of T&CAM and conventional medicine for cancer treatment. Therefore, when establishing a treatment guide for cancer patients and conducting a national R&D, the combination of T&CAM and conventional medicine should be considered. Policy support (e.g., health insurance) is needed to provide evidence-based cancer treatment to patients based on clinical evidence.

Based on the results, we suggest the following for future studies: (i) Surveys, systematic reviews, retrospective studies, and clinical studies are needed to investigate which T&CAM treatments are frequently used for specific cancers; (ii) Multicenter and international RCTs are necessary to clearly determine the point of intervention for T&CAM by comparing T&CAM treatment with conventional medicine for specific cancers; (iii) The studies need to be conducted with suitable methods and data for each country in the United States, Europe, and other regions.

## Conclusion

Our study findings showed that cancer occurrence within an individual did not lead to the use of T&CAM alone but led to the simultaneous use of conventional medicine and T&CAM. Among participants already using T&CAM, the occurrence of cancer significantly increased the number of T&CAM outpatient visits. It was also found that T&CAM has been utilized more often by the most vulnerable people, such as medical beneficiaries and those with a low level of education.

## Data Availability

The Korea Health Panel (KHP) data used in this study can be obtained at https://www.khp.re.kr:444/eng/data/data.do after registration.

## Abbreviations

EQ-5D: EuroQol-5 Dimension
T&CAM: Traditional, complementary and alternative medicine
KHP: Korea Health Panel
QOL: Quality of life
HRQOL: Health-related quality-of-life
KIHASA: Korea Institute for Health and Social Affairs
OR: Odds ratio
